# Public Perceptions around mHealth Applications during COVID-19 Pandemic: A Network and Sentiment Analysis of Tweets in Saudi Arabia

**DOI:** 10.3390/ijerph182413388

**Published:** 2021-12-20

**Authors:** Samar Binkheder, Raniah N. Aldekhyyel, Alanoud AlMogbel, Nora Al-Twairesh, Nuha Alhumaid, Shahad N. Aldekhyyel, Amr A. Jamal

**Affiliations:** 1 Medical Informatics and E-learning Unit, Medical Education Department, College of Medicine, King Saud University, Riyadh 12372, Saudi Arabia; sbinkheder@ksu.edu.sa (S.B.); raldekhyyel@ksu.edu.sa (R.N.A.); 2 Freelance Research Assistant, Riyadh 12372, Saudi Arabia; AlAnoud.Saud94@gmail.com; 3 Information Technology Department, College of Computer and Information Sciences, King Saud University, Riyadh 12372, Saudi Arabia; twairesh@ksu.edu.sa; 4 STC’s Artificial Intelligence Chair, King Saud University, Riyadh 11451, Saudi Arabia; 5 College of Public Health and Health Informatics, King Saud bin Abdulaziz University for Health Sciences, Riyadh 14611, Saudi Arabia; humaidn@ksau-hs.edu.sa (N.A.); shahadaldekhyyel@gmail.com (S.N.A.); 6 Evidence-Based Health Care & Knowledge Translation Research Chair, King Saud University, Riyadh 11451, Saudi Arabia; 7 Family & Community Medicine Department, College of Medicine, King Saud University, Riyadh 12372, Saudi Arabia

**Keywords:** COVID-19, coronavirus, social media, Twitter, mHealth applications, public health, sentiment analysis, network analysis, health informatics

## Abstract

A series of mitigation efforts were implemented in response to the COVID-19 pandemic in Saudi Arabia, including the development of mobile health applications (mHealth apps) for the public. Assessing the acceptability of mHealth apps among the public is crucial. This study aimed to use Twitter to understand public perceptions around the use of six Saudi mHealth apps used during COVID-19: “Sehha”, “Mawid”, “Sehhaty”, “Tetamman”, “Tawakkalna”, and “Tabaud”. We used two methodological approaches: network and sentiment analysis. We retrieved Twitter data using specific mHealth apps-related keywords. After including relevant tweets, our final mHealth app networks consisted of a total of 4995 Twitter users and 8666 conversational relationships. The largest networks in size (i.e., the number of users) and volume (i.e., the conversational relationships) among all were “Tawakkalna” followed by “Tabaud”, and their conversations were led by diverse governmental accounts. In contrast, the four remaining mHealth networks were mainly led by the health sector and media. Our sentiment analysis approach included five classes and showed that most conversations were neutral, which included facts or information pieces and general inquires. For the automated sentiment classifier, we used Support Vector Machine with AraVec embeddings as it outperformed the other tested classifiers. The sentiment classifier showed an accuracy, precision, recall, and F1-score of 85%. Future studies can use social media and real-time analytics to improve mHealth apps’ services and user experience, especially during health crises.

## 1. Introduction

The novel coronavirus disease (COVID-19), caused by severe acute respiratory coronavirus 2 (SARS-CoV-2 virus), has spread around the world causing a pandemic. In Saudi Arabia, the first COVID-19 confirmed case was reported on 2 March 2020, which was followed by a series of mitigation efforts imposed by the government. These efforts included the enforcement of social distancing, closure, and suspension of schools and universities, shopping malls, restaurants, coffee shops, public parks, sports leagues and competitions, and congregational and weekly Friday prayers [1]. On 23 March 2020, the Saudi Arabian government took extra measures by announcing a national wide curfew, which lasted about two months [2,3]. During the implementation of these precautionary measures, communication technology tools and social media platforms were among the main methods that authorities used to communicate with the public. The development of specific mobile health applications (mHealth apps) for public use was also a major pandemic response by the Saudi government [1,4].

The importance of using mHealth apps for improving public health and transforming health service delivery has been recognized by the world health organization (WHO) since 1998 [5,6,7]. The Saudi Arabian national e-health initiative has also recognized the importance of e-health by mentioning e-health as an enabler of quality and safe healthcare systems [8]. During the COVID-19 pandemic, and as a response to the public health crisis, many governments leveraged technologies that played a role in combating the COVID-19 pandemic. Such technologies were focused on developing e-health applications, which included the use of mobile integrated health care programs from home, mHealth apps, artificial intelligence (AI) and machine learning decision-making apps, robotic technologies, social networking apps, contact tracing apps, AI, and blockchain-enabled decentralized apps, and health and fitness apps [4,9,10,11,12,13].

The Saudi government, in collaboration with specialized organizations, has launched six mHealth apps, which were heavily used during the pandemic [12,14]. These mHealth apps were the official apps providing free services to the public. There were three mandated apps used for COVID-19 testing, isolation, and issuing electronic permits for movement, gathering, and work [14]. Three of the mHealth apps were specifically designed in response to the pandemic during the year 2020: “Tetamman” [15] (translated to English as “rest assured”), was launched in April 2020, “Tawakkalna” [16] (translated to English as “we trust”), launched in May 2020, and “Tabaud” [17] (translated to English as “social distancing”) was launched in June 2020. The remaining three mHealth apps were developed before the pandemic, which were designed to support telemedicine services and primary health clinic appointment scheduling: “Sehha” [18] (translated to English as “health”), launched in March 2017, “Mawid” [19] (translated to English as “appointments”), launched January 2018, and “Sehhaty” (translated to English “my health”), was launched in August 2019 [4,14,20]. However, some research studies reported usability barriers among mHealth apps users during the COVID-19 pandemic, including lack of knowledge, awareness, trust, and lower users’ satisfaction [12,21,22,23]. Therefore, a critical component for enhancing the meaningfulness of the implemented mHealth apps during the COVID-19 pandemic is to understand perceptions, experiences, and acceptance among their users [24,25].

Social media platforms are a great resource to collect information regarding user experiences and perceptions due to their popularity. Many people use the platform as a method to share their opinions, experiences, and ideas, especially during public health crises [26,27,28,29,30,31]. One of the most popular social media platforms is Twitter. Twitter can be seen as an important resource, which may be used by consumers to seek health-related information, engage in behavior change interventions, and share perceptions, and by researchers and health officials to track disease outbreaks and drug use [32,33,34,35]. For example, an infoveillance approach was used to support public health decision-makers during the 2009 H1N1 pandemic by providing near real-time content and sentiment analysis [36]. Furthermore, researchers have identified health-related keywords and hashtags, which can be used in analyzing tweets during public health pandemics or outbreaks. Signorini et al. collected tweets matching a set of 15 pre-specified search keywords including “flu”, “vaccine”, “tamiflu”, and “H1N1” and built a predictive model based on 1 million influenza-related tweets [33]. Using social media and understanding public conversations can help in gaining insights into the impact of various implemented measures during a crisis, including the use of eHealth and mHealth apps.

Social network analysis and sentiment analysis from social media data have also played a significant role in supporting stakeholders, such as governments, health authorities, and policymakers, in data-driven decision making during pandemics and outbreaks for timely responses during public health emergencies [26,27,28,29,30,31]. Social network analysis is an interdisciplinary research area that examines information flow, attitudes, and patterns gained from exchanged conversations and users characteristics [37]. For instance, Park et al. investigated information-sharing patterns during the COVID-19 pandemic by applying a network and content analysis of four networks, which suggested that the spread of information was faster in the Coronavirus network than in others [38]. Similarly, sentiment analysis can help decision-makers in understanding the sentiments of people about topics, such as medical information and public health, and to improve healthcare services [39].

Several studies applied either social network analysis or sentiment analysis to explore public perceptions toward some health-related topics, such as COVID-19 pandemic [40,41], 5G COVID-19 conspiracy theory and misinformation [31], vaccination [42,43], child physical activity [44], quality of care [45], and end-of-life care [46]. Studies that combined methodologies of social network analysis and sentiment analysis were generally lower than studies that used either social network analysis or sentiment analysis. For instance, Shams et al. experimented with the combination of sentiment analysis and social network analysis in building classification rules to represent customers’ preferences and needs and found that this combination helped in classifying products based on customers’ interests [47]. Hung et al. analyzed Twitter discussions and the related sentiments toward COVID-19 and concluded that Twitter discussions and sentiments can help officials with needed information during pandemics [35]. Furthermore, Yao et al. also applied both social network analysis and sentiment analysis to the construction safety research among the public [48].

At the time of this study, there was no research in Saudi Arabia that examined public perceptions about the use of mHealth apps during COVID-19 by probing Twitter data. Even though some published research studies have evaluated the perceptions of users on the use of mHealth apps during the pandemic, these studies relied only on surveys [23,49,50]. Unlike social media-based data collection, traditional survey-based data collection might suffer from a tendency to systematic bias due to underrepresenting the sample or fall into a systematic bias due to the survey design. Furthermore, surveys require individuals to recall their experiences and sentiments regarding a specific context, while social media collects data from real-time and real-world individual interactions on a larger scale [51]. Lastly, using the conjunction of social network analysis and sentiment analysis has not experimented with the context of mHealth apps. Therefore, understanding how these methods can help in gaining insights about users’ experiences from Twitter data is beneficial to improve the usability of mHealth apps.

Therefore, the aim of this study is to use Twitter as a source of data, to understand conversations and perceptions of users around the use of six mHealth apps during the COVID-19 pandemic by conducting a network and sentiment analysis of tweets. The specific objectives of this study are: (1) to examine the difference in communication network structure across the networks generated among the six mHealth apps included in our study; (2) to analyze the sentiment surrounding the six mHealth apps conversations; and (3) to evaluate the performance of a sentiment classifier using machine learning approaches.

## 2. Materials and Methods

### 2.1. Saudi’s mHealth Apps

The six mHealth apps (Table 1) included in our study were based on published research describing the country’s digital response to the COVID-19 pandemic [4,14,20]. These mHealth apps were used for (1) telehealth (“Sehha”), (2) digital screening (“Mawid”, “Sehhaty”), (3) follow-up (“Tetamman”), (4) Current health status and permits (“Tawakkalna”), and (5) COVID-19 contact notification (“Tabaud”).

“Sehha” is a telehealth app that provides online medical consultations, including audio-video medical consultations, AI technologies, and health assessment tools. The application “Mawid” is an e-appointment app used for scheduling appointments at one of the Ministry of Health (MOH)’s primary care centers and hospitals. During the pandemic, “Mawid” was used as a tool for virtual COVID-19 screening. The application “Sehhaty” provides e-health services and health information access, such as vital science, steps tracker, medications, and sick leaves. “Sehhaty” app was also used as a symptom checker tool and for scheduling COVID-19 tests either at Tetamman clinics (which differs from the Tetamman app) or drive-thru locations. “Tetamman” app is a preventative technological solution during the COVID-19 pandemic, which is mainly used during domestic isolation or quarantine based on exposure, such as travel. “Tetamman” app provides several services, including COVID-19 test results, symptoms check-up, educational content library, countdown indicator for isolation days, and alerts. The application “Tawakkalna” is a GPS-enabled app, which provides several services, including the COVID-19 latest health status (e.g., no record of infection, infected, exposed, arrived from abroad, and immune), requesting movement permits during curfew, and notifying the user of close contact of an infected person or any isolated areas. “Tabaud” app relies on Bluetooth technology to limit the spread of COVID-19 and notify users when exposure is detected. “Tabaud” helps in notifying people that came in contact with confirmed COVID-19 cases. People with confirmed COVID-19 tests can voluntarily share their results with people whom they had contacted during the past 14 days through the app.

### 2.2. Study Design and Data Collection

**Figure 1:**
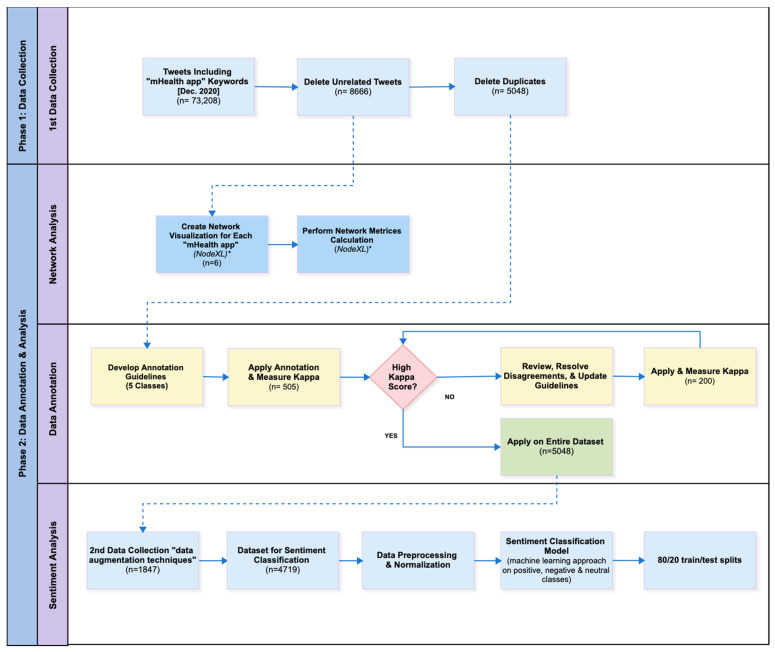
Two-phase study methodology: “Phase 1: Data collection” and “Phase 2: Data annotation and analysis”, including network and sentiment analysis. * NodeXL is a network analysis and visualization software package for Microsoft^®^ Excel^®^.

Our study involved a retrospective analysis of public conversations posted on Twitter [52], which mentioned one or more of the six mHealth apps during COVID-19. This study used two main methodological approaches: network analysis and sentiment analysis (Figure 1).

#### 2.2.1. Data Collection for Network Analysis

Data were retrieved on 12 December 2020, using the Twitter search API embedded in NodeXL (Social Media Research Foundation) [53], a network analysis and visualization software package for Microsoft^®^ Excel^®^ that also provides a set of import tools to collect data from Twitter and other social media accounts. We retrieved Twitter users with recent tweets containing the keywords listed in Appendix A. These keywords represent terms related to mHealth apps used during COVID-19 in Saudi Arabia. Twitter limits the number of retrieved tweets to a maximum of 18,000 tweets per hour. With this limitation, a total of 73,208 tweets were retrieved. Appendix A describes the number of tweets included in our study data set. Data ranged from 9 April 2020 to 8 December 2020. We then manually reviewed the tweets to include those with the terms related to the mHealth apps for further analysis. Our final mHealth app networks consisted of a total of 4995 Twitter users and 8666 conversational relationships, which included “tweets”, “retweets”, “replies”, and “mentions”.

#### 2.2.2. Data Collection and Annotation for Sentiment Analysis

We removed duplicate tweets based on the “unified Twitter ID” (a unique ID generated from Twitter associated with every single tweet), resulting in a total of 5048 tweets. To understand public opinions about the six mHealth apps, two annotators (S.B. and A.A.) manually annotated all the tweets in the data set. There were five annotation classes: (1) positive when the text expressed a positive opinion, (2) negative when the text expressed a negative opinion, (3) neutral when the text did not include any opinion such as facts and inquiries, (4) indeterminate when there was an opinion but there was unclear polarity or was unclear if there was an opinion [54], and (5) sarcasm when an opinion expressed the opposite in a sarcastic form and usually a negative opinion. Intercoder reliability scores were calculated using Cohen’s kappa, which is a statistical measure used for interrater reliability testing with the capability to account for chance agreement [55]. The ‘Kappa.test’ function in the ‘fmsb’ package [56] part of the statistical software R (version 4.0.3) was used [57]. There was a strong agreement between the two annotators over the five annotated classes (κ = 0.86, *p* < 0.001). Disagreements in annotations were resolved by discussion to reach a consensus between the two annotators. The final counts of tweets based on the five sentiment classes are shown in Table 2. After the annotation was completed, tweets that were labeled as indeterminate and sarcasm were excluded from the data set since they are expected to cause ambiguity for the machine learning classifier.

To build the sentiment classifier, classes within the data set should be balanced. However, we observed a high imbalance between the positive, negative, and neutral classes after completing the annotation in the initial data set. Therefore, data augmentation techniques were followed, which ensure the presence of sentiment polarity (positive, negative) in the tweets. This was accomplished by using a list of positive and negative Arabic words that were combined with the mHealth apps’ keywords shown in Appendix A and included as the search query using the Twitter search API. A total of 1847 additional tweets were collected for annotation. Having a high kappa score in the initial data set (Table 2), the additional set of tweets was annotated by one annotator (A.A.).

The final data set is shown in Table 3 after removing duplicate tweets and the indeterminate and sarcasm classes. The final data set was used to serve two purposes: first, to analyze the sentiment of each mHealth app using the human-in-the-loop approach, which is the process of integrating human knowledge and experience to facilitate or improve a prediction model; second, to train a machine learning classifier, which is an automated approach that inputs the training examples that were annotated by the humans and outputs the predicted classes [58], using the positive, negative, and neutral instances. Visualization was performed using Tableau Desktop (version 2021.4.1) [59].

### 2.3. Data Analysis

#### 2.3.1. Social Media Network Analysis

A social network is composed of nodes and links where nodes represent users and edges represent conversations or interactions between users. We compiled users by subgroup using the Clauset–Newman–Moore cluster algorithm [60], and we visualized the networks using the Harel–Koren Fast Multiscale layout algorithm [61], which are commonly used in social communication research [31,38]. We created a network for each of the six mHealth apps, then calculated metrics for each network. To identify the influencing Twitter accounts in these conversations, the nodes were ranked using two measures: “betweenness centrality”, which is a measure of importance, and “PageRank”, which is a measure of influence [62]. We only included top Twitter accounts per measure and did not include personal accounts due to Twitter users’ privacy policies. All the tasks for the network analysis, including the calculation of network metrics and the visualization of networks, were performed using NodeXL [53].

#### 2.3.2. Sentiment Classification

Sentiment classification is a text classification task that classifies text into polarity (positive, negative, neutral). The typical pipeline for such a task is text preprocessing, feature extraction, then the data and features are fed into the Machine Learning (ML) classifier. This is the conventional ML approach. With the recent abundance of textual data on the Web, deep learning approaches have been introduced, including context-independent models to represent words such as the Word2Vec model [63] and the context-dependent transformer-based models such as Bidirectional Encoder Representations from Transformers (BERT) [64]. These natural language processing models have the advantage that they do not require manual feature engineering (hand-driven features), and features can be learned automatically from the text. Therefore, to construct the sentiment classifier in this study, we experimented with three different approaches: classical ML with Term Frequency-Inverse Document Frequency (TF-IDF), Word2Vec model, and BERT.

The final data set (Table 3) was used to construct the sentiment classification model. First, the data set was preprocessed by removing URLs, mentions, and English characters using the natural language toolkit (NLTK), regular expression (regex), and the “genism” Python libraries. Arabic letter normalization was also performed. Then, the data set was split into 80/20 train/test splits.

For classification, we used the Support Vector Machine (SVM), one of the most used algorithms by researchers, and proved to provide high accuracy for Arabic sentiment classification [65]. The conventional TF-IDF, which relies merely on simple counts, was used in the first experiment with the SVM classifier. Then in the second experiment, we used the Word2Vec embeddings of AraVec [66] as features to represent the text of the tweets. Specifically, we used the version of AraVec that was constructed from Arabic Tweets using the Skip-gram model with dimension = 300. For the third experiment, we used AraBERT [67], an Arabic pre-trained language model. This experiment was performed using an Adam optimizer with a learning rate of 2 × 10−6 and a batch size of 8 for 10 epochs. The max sequence length was set to 256. The finetuning was performed by adding a softmax classification layer to the pre-built sentiment classification model.

## 3. Results

### 3.1. Comparing mHealth Conversations Networks

**Figure 2:**
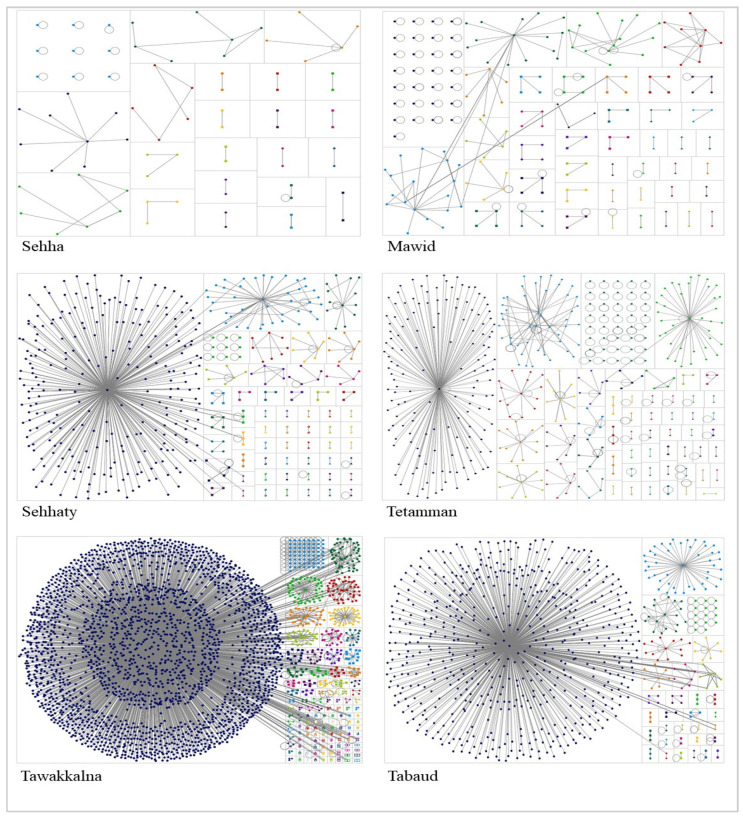
The six mHealth apps networks using Twitter conversations. “Sehha” (top left), “Mawid” (top right), “Sehhaty” (middle left), “Tetamman” (middle right), “Tawakkalna” (bottom left), and “Tabaud” (bottom right).

The topologies of the six networks are shown in Figure 2. The networks showed that the patterns of conversations (edges) between users (nodes) were similar across the users of the following three mHealth apps: “Sehhaty”, “Tawakkalna”, and “Tabaud”. Each of “Sehhaty”, “Tawakkalna”, and “Tabaud” network has a large cluster (community) that was connected with central nodes: Saudi Ministry of Health “@SaudiMOH” for “Sehhaty”, “@Tawakkalnaapp” for “Tawakkalna”, and “@TabaudApp” for “Tabaud”. Appendix B displays the top accounts that were found in the conversations related to the mHealth apps that were included in this study, ranked by two measures: “betweenness centrality” and “PageRank” [62]. Users with a high “betweenness centrality” score indicate that their position within the network, which is typically government accounts, allows them to become the gatekeeper or link between the communities [68]. “PageRank” high scores indicate that these users are of high importance and high influence. The users with the highest “betweenness centrality” and “PageRank” scores in “Tawakkalna” and “Tabaud” were found to be associated with education officials (e.g., ministry of education and universities), governments, hajj and umrah, media, and health-related governments or hospitals. In comparison, users with the highest “betweenness centrality” and “PageRank” scores in “Sehha”, “Mawid”, “Sehhaty”, and “Tetamman” were found to be majorly associated with media and health-related governments or hospitals accounts.

Table 4 shows the conversational relationship characteristics among users within each network. Unique edges mean fewer overlapping relationships, which reflect an instant community with many one-time conversations [38]. The network with the highest frequency of unique edges was “Sehha” followed by “Mawid”, then “Tetamman”. The networks with high frequencies of edges with duplicates indicate that conversations were continuously and more frequently exchanged among users. These networks were “Tabaud”, followed by “Tawakkalna” then “Sehhaty”.

Self-loops, known as a conversation thread starting and ending with the same user [38], was found to be more frequent in “Mawid”, followed by “Tetamman” then “Sehha” networks. The lowest frequencies of self-loops were observed in “Tawakkalna”, “Sehhaty”, and “Tabaud” networks indicating more interactions with others in conversations. “Mawid” network also had the largest percentages of isolates, users with zero connections, followed by the “Sehha” network.

Table 5 summarizes the overall metrics of each network. The “geodesic distance” is the value that reflects the shortest path between two users (nodes); also, within a network, the maximum geodesic distance (diameter) is the furthest distance [38]. “Sehha”, “Tetamman”, then “Tabaud” networks have the smallest diameter, which might indicate that users involved in these conversations communicated more frequently with each other with a faster spread of information than other networks. “Tawakkalna” and “Sehhaty” networks have the largest diameter. For the component analysis, which means that for every pair of users, there is a path, the results show that users from “Tawakkalna” had the largest numbers of connected components. “Tawakkalna” and “Tabaud” have the “largest chat rooms” [38] with the largest number of users (nodes) in a connected component and maximum edges in a connected component, followed by “Sehhaty”.

Modularity is “a measure of the network structure designed to measure the strength of division of a network into modules” [69]. The highest modularity value indicates that the strength of the connection within a sub-network (community A) is higher than across sub-networks (community B). Low modularity values indicate that the clusters are well-defined and that the users within the clusters rarely move to another cluster [38]. The modularity values of the “Sehha” and “Mawid” networks were the highest among the networks; in contrast, the modularity values of the “Tawakkalna” and “Tabaud” networks were the lowest among other networks.

### 3.2. Sentiment Analysis of Conversations Surrounding mHealth Apps

The manual annotation resulted in identifying conversations associated with positive, negative, or neutral sentiments (Table 2). Tweets with a positive sentiment focused on appreciation, positive opinions, and expressions around government trust. Negative tweets covered weaknesses, issues faced with apps, negative opinions, and negative psychological impact (Appendix C). Neutral tweets included facts or information pieces, neutral suggestions, and general inquires. Table 6 shows examples of positive, neutral, and negative sentiments per each mHealth app. As we evaluated the sentiment of tweets related to these networks, we found that most tweets were neutral and focused more on users seeking information and asking questions about the apps rather than providing their opinions. Within each mHealth app sentiment, the highest frequency of positive opinions was found for “Tetamman” (35.9%). The mHealth apps that had more positive sentiments than negative were: “Sehha”, “Tetamman”, and “Tabaud”. The mHealth apps that had more negative than positive sentiments were: “Mawid”, “Sehhaty”, and “Tawkkalna”.

### 3.3. Performance of an Automated Sentiment Classifier

**Figure 3:**
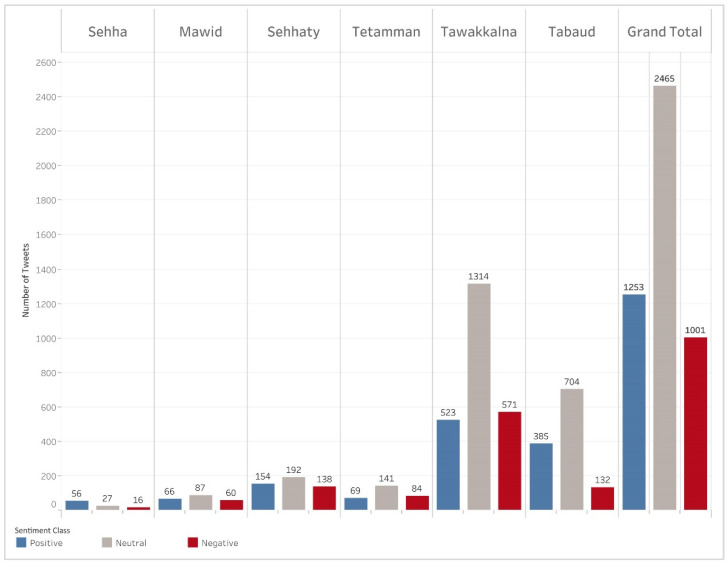
The sentiment of each mHealth app after data augmentation. These tweets were used for the sentiment classifier.

Figure 3 shows the number of tweets after data augmentation for the sentiment classifier, which included unique tweets after processing. There was an increase in the numbers of positive and negative tweets across all apps except “Tetamman”. The app “Tawakkalna” remained the same, with more negative tweets than positive. Table 7 shows the performance of the three classifiers, as explained in Section 2.3.2. We observed that the best performing classifier was the SVM with AraVec embeddings (F1-score = 0.85) in contrast to SVM tf-idf (F1-score = 0.84) and AraBERT (F1-score = 0.80).

## 4. Discussion

### 4.1. Major Findings

Our study presented a novel research context by using social media conversations posted on Twitter to assess public perceptions on using mHealth apps during the COVID-19 pandemic. Two methodological approaches were used, which are the social network analysis and the sentiment analysis. Twitter data were used to identify the networks and sentiments of the public toward six mHealth apps, which were “Sehha”, “Mawid”, “Sehhaty”, “Tetamman”, “Tawakkalna”, and “Tabaud”. The social network analysis identified similar patterns in conversations among “Sehhaty”, “Tawakkalna”, and “Tabaud”. On the other hand, similar patterns were found among the following networks: “Sehha”, “Mawid”, and “Tetamman”. The apps “Tawakkalna” and “Tabaud” were the largest networks in size (the number of users) and volume (the number of conversational relationships) among all, and their conversations were led by a variety of governmental accounts. In comparison, the apps “Sehha”, “Mawid”, “Sehhaty”, and “Tetamman” networks were mainly led by a health sector or/and media. The sentiment analysis showed that conversations around the six mHealth apps were majorly neutral. Among all the six mHealth apps included in this study, we found that conversations about “Tetamman” were the highest frequency in positive sentiments. For the automated sentiment classifier, we used the SVM with AraVec embeddings as it outperformed other tested classifiers. The sentiment classifier showed an accuracy, precision, recall, and F1-score of 85%.

Overall, the social network analysis identified similar patterns in conversations among “Sehhaty”, “Tawakkalna”, and “Tabaud”. These mHealth apps had the highest number of conversations, indicating their significant role during the pandemic, and were heavily used by the public for COVID-19 health status, tracing of cases, and exposure notifications. A previous study has also reported that “Tawakkalna” and “Tabaud” mHealth apps were among the highest in the number of users during the pandemic [12]. The fact that “Tawakkalna” and “Tabaud” had distinguished Twitter user accounts while other apps did not might have also contributed to the highest number of conversations surrounding them. When examining the main conversational role-players within the networks, conversations around “Tawakkalna” and “Tabaud” were led by various governmental accounts, including education, hajj and umrah, the health sector, and media channels. This variability is most likely a result of the apps’ medical-related features and the regulations enforced by the Saudi government to combat the spread of COVID-19 through the mandatory use of “Tawakkalna” when entering universities, hospitals, workplaces, shopping malls, government buildings, and other public places.

When compared with other networks featured that had more app functionalities, the conversations of “Sehha”, “Mawid”, and “Tetamman” networks lacked interactions. A possible reason for low interactions and conversations in Twitter around “Sehha” and “Mawid” apps may be the misperceptions among the public regarding the access to MOH’s apps for only MOH patients [23]. Another reason for low interactions, some studies that surveyed physicians about their perspective on telehealth during the pandemic showed that they were concerned about the following: technological barriers, diagnostic reliability, cultural and social factors, lack of face-to-face interactions, and lack of a clear telemedicine legal framework. In addition, physicians tend to use WhatsApp^®^ and Zoom more than the “Sehha” app [50,70,71]. Therefore, more campaigns targeting the eligibility of these mHealth apps are suggested to increase awareness about their use [23]. Lastly, even though “Tetamman” was one of the mHealth apps that were launched during the pandemic, the conversations were not as extensive as those by “Tawakkalna” and “Tabaud”. A plausible reason for this low rate of conversations is that many of the services provided by “Tetamman” were already offered by “Sehhaty” and “Tawakkalna”. This has also been found in our sentiment analysis findings where users suggested a need to integrate mHealth apps into one fully featured app.

Several findings of this study were derived from the sentiment analysis of conversations around the use of the six mHealth apps. First, the majority of conversations around mHealth apps were neutral. The dominance of neutral tweets was also reported in other similar studies depending on the research topic domains, where some topics can be more controversial than others [72,73,74]. The neutral conversations provided information or facts, neutral suggestions, and general inquires, which may indicate that Twitter can be used as an effective real-time communication platform to answer users’ questions and tackle their concerns. This is in line with many other studies that showed the use of Twitter by government officials during pandemics to communicate with the public during health crisis times [30,75,76,77]. Second, the findings also indicated several positive conversations that were relevant to the appreciation of the mHealth apps’ services and features, in addition to the positive user experiences surrounding the use of these apps. Other positive conversations were more of statements that indicated gratitude and appreciation toward healthcare providers. Many of the communication campaigns on social media platforms, and other communication outlets, which were led by the MOH during the pandemic, were focused on lifting the spirits of the public in the fight against the pandemic.

Third, when examining the type of issues raised by the public indicated by the negative sentiments, several were related to the recent digital transformation of many Saudi government services and the adaptation of mHealth and eHealth apps to facilitate health-related services, as stated by Han et al. [78]. Concerns and lack of familiarity and digital literacy by the public are expected at these early stages of adaptation [79,80]. Furthermore, the replication and overlapping of features between the mHealth apps have been a concern that was raised frequently by the public. Such duplication in services should be avoided as it could lead to confusion and avoidance of using these mHealth apps altogether, which may have contributed to the negative experience. Integrating similar features between these mHealth apps into one app may overcome these issues. Other negative conversations were related to the technical and accessibility issues experienced by users. All the mHealth apps described in our study require the use of Wi-Fi or a cellular connection, an issue with mHealth apps in general [81]. It is vital to consider this limitation when mandating the public to use a specific mHealth app, given the variability and differences in the availability of smartphones and Internet connections among the public. Another critical element raised in these conversations was the psychological impact that may be related to the use of such apps. The use of mHealth apps to track COVID-19 cases and their negative implications on the public has been a topic addressed by many researchers. Examples of these implications include increased levels of anxiety when users receive a COVID-19 exposure notification [81,82,83]. Privacy concerns have also been raised about tracking and tracing features, specifically about “Tetamman”, “Tawakkalna”, and “Tabaud”, similar to what has been reported by other mHealth apps [84,85,86]. The benefits and drawbacks of mHealth systems that raise issues with consumer privacy, must be examined critically by all stakeholders to ensure public by in and trust is not jeopardized.

To build the sentiment classifier for our data set, we experimented with the performance with different approaches. Overall, the results showed that AraVec embeddings performed better than AraBERT. This might be because AraVec embeddings were pre-trained on tweets compared to AraBERT that was pre-trained on Arabic Wikidumps and other Arabic corpora. Unlike different text sources on the Web, the nature of text in tweets is known to be informal with different characteristics. Consequently, the SVM with word embeddings sentiment classifier performed well, and it can be used in automating the detection of the sentiment of conversations around mHealth apps.

### 4.2. Theoretical Contributions

Social network theory, “a set of general assumptions about a domain of study and the methods suitable for its investigation”, is concerned about the relations between individuals who create, share, and disseminate knowledge as a unit of analysis [87]. The social network theory contributes to building explanations from patterns of social relations between individuals within online communities [88], where online social interactions are very similar to face-to-face communities [89]. Moukarzel et al. stated that the analysis of networks combined with other methods can provide opportunities in gaining a deeper understanding of real-time interactions and relationships among the public within a certain context [89]. This study illustrated that there is value gained from integrating and combining social network analysis with sentiment analysis to the context of mHealth apps. This integration has the potential in enabling researchers to evaluate behaviors among the public surrounding the use of mHealth apps real-world discussions.

Twitter is a potential source for big data. Unlike the traditional use of Twitter analytics (e.g., number of likes and retweets), recent directions grounded in social network theory enabled the analysis of Twitter data in robust, meaningful, and theoretically grounded methods [89]. The relationship of usability and sociability [90] within the context of mHealth app online communities is new. This relationship provides an opportunity to understand users’ needs, which may improve the usability, design, and functionalities of mHealth apps. The knowledge gained in this study has also enabled us to identify some practical strategies and recommendations (Section 4.3.) to support and improve the mHealth apps and their online communities. Unlike other cross-sectional studies that focused on collecting users’ perceptions through a survey tool [23,49,50], this study used social media as it reflects real-world interactions among people when a real-time response is needed.

### 4.3. Practical Implications

The study findings showed that assessing the perceptions of users surrounding the use of mHealth apps during the COVID-19 pandemic through analyzing public opinions posted on social media accounts is crucial in providing direction for future health crises and improving mHealth app services. This research supports the fact that the development of mHealth technologies requires collaborative efforts between end-users and implementers to meet person-centered needs and improve the users’ experiences in different ways. On a broader scale, health authorities and organizations can implement real-time social network analysis and sentiment analysis to automate the analysis of public perceptions and opinions related to mHealth apps. By using social networks and sentiment analyses, the results of this study provide insights into public behavior and acceptability toward the six mHealth apps included in our study. Box 1 shows a list of recommendations and lessons learned based on our study’s analyses of Twitter conversations around six mHealth apps that can be used to improve mHealth services during a pandemic.

Box 1 **A list of recommendations and lessons learned that can be used to strategically improve mHealth apps services during a pandemic.**

- Using social media data as a source and a connection tool for understanding public perceptions, opinions, and acceptability around mHealth apps can serve as a real-time communication approach during pandemics to answer questions of the public and tackle users’ concerns;
- Health authorities and organizations can implement real-time sentiment classifiers to automate the analysis of public perceptions and opinions about mHealth apps;
- Establishing coordinated efforts among governmental entities in developing public mHealth apps, guided by the country’s digital health strategy, may have the potential to increase a positive user experience and lessen the negative experience associated with the use of mHealth apps during a pandemic;
- Increasing campaigns targeting the public regarding mHealth apps is suggested to increase awareness about these apps;
- Having an official Twitter account associated with a mHealth app, which is led by experts, is recommended to engage the public in conversations related to the use of the app and would serve as a platform for information distribution;
- Enhancing mHealth apps with pandemic-related information and services may increase their use by the public (e.g., telemedicine, COVID-19 testing, health status, vaccination updates, and contact notifications);
- Avoiding duplicate features among apps or similar app names by integrating mHealth apps with similar features into one app may increase the use among the public and positive experiences;
- For mandated mHealth apps, app developers should consider people with limited access to Internet services, thus providing the features of the app offline;
- Negative sentiments are likely to be driven by psychological impact, lack of familiarity and digital literacy, and technical and accessibility issues. Such sentiments may be alleviated by considering different age groups, increasing accessibility, designing educational material, and creating connection channels with the public to address their concerns;
- Governmental communication efforts toward non-English expatriates were seen by the MOH, given that the cooperation of expatriates living in Saudi Arabia played an important role in COVID-19 mitigation and control measures. Designing mHealth apps in different languages other than English and Arabic may enhance the positive user experience among this population.

### 4.4. Limitations and Future Research

This work does not stand without limitations. First, the Twitter API limits the number of tweets that were retrieved. However, the API limits did not affect the applicability and performance of the sentiment classifier. Second, the extracted Twitter data might reflect Twitter users, but not all where young users tend to use Twitter more than the elderly [23,38]. Third, we extracted data from the dominant social media platform in Saudi (Twitter). Other platforms such as Instagram and Facebook may have included other sentiment topics, which were not captured in our study. Lastly, this study focused on a specific mHealth app population; users who posted their opinions on Twitter in the Arabic language. Other users who posted their opinion in English or used mHealth apps developed by private entities may have different opinions regarding the use of mHealth apps during COVID-19.

There are several opportunities for future research. First, future studies can combine more than one analytical approach to analyze users’ opinions by combining multiple data sources, such as surveys and social media. For example, surveys can be administered to mHealth apps’ users to evaluate the usability of these applications, demographic factors, and the challenges that users might face. A similar approach was used by Twitter polls to gain public insights on telemedicine [91] and can be applied to other mHealth apps. Second, future analyses can include topic modeling or content analysis for further quantitative understanding of the discussed topics, concerns, and perceptions. For instance, it can focus on standardized content analysis to assess the positive and negative tweets and explore how negative sentiments can be alleviated. Third, sentiment analysis can be combined with other Twitter metrics, such as tweets’ likes, retweets, and followers for each topic, to calculate the sentiment interaction rate [28] for each mHealth apps. Fourth, conducting an in-depth analysis of mHealth public perceptions by aggregating Twitter users’ sentiments to identify factors that might have driven the opinion change of users and identify communication structures, styles, or sources that increase positive sentiment or alleviate concerns. Fifth, future studies may focus on conducting social network and sentiment analysis centering on the general community’s sentiment by excluding tweets posted by key nodes, such as the apps’ official Twitter accounts and or governmental entities. Finally, a future study can focus on measuring the perceptions of the public around mHealth apps using similar approaches discussed in this study on a larger scale of social media data.

## 5. Conclusions

This study showed that social media could be used as a complementary data source and a connection tool to improve user experience and to understand public perceptions about the use of mHealth apps during a pandemic. Furthermore, there is value gained from integrating and combining social network analysis with sentiment analysis in the context of mHealth apps to enhance the understanding of their usability from real-world discussions. The network analysis showed that “Sehhaty”, “Tawakkalna”, and “Tabaud” had similar network patterns with more interactions in conversations than other networks. “Tawakkalna” and “Tabaud” were the most extensive networks among all. The sentiment analysis approach showed that most Twitter conversations around the six mHealth apps were neutral. The sentiment classifier performed well. Therefore, health authorities and organizations can implement real-time sentiment classifiers to automate the analysis of public perceptions and opinions about mHealth apps. In addition, it is imperative for developers to decrease the number of apps with shared or similar functionalities as it might lead to user confusion. Efforts should focus on enhancing positive experiences with these mHealth apps, which would hopefully lead to increased positive opinions reflected in social media conversations. Having distinguished Twitter user accounts for high-impact mHealth apps is recommended, as it provides quick access for apps’ users to express their opinions, concerns, inquires, and recommendations. What is even more important is for these accounts to be actively led by experts who interact with the public and facilitate their adaptation of such mHealth apps. The list of recommendations and lessons learned derived from Twitter conversations around six mHealth apps in this study can be used to strategically improve the user experience of the mHealth apps during pandemics.

## Figures and Tables

**Table 1:** Description of the six mHealth apps used in response to the COVID-19 pandemic in Saudi Arabia.

mHealth App	Translation in English	COVID-19 Primary Use	Mandatory	Existed before COVID-19
Sehha	Health	Telehealth	No	Yes
Mawid	Appointments	Digital screening ^1^	No	Yes
Sehhaty	My Health	Digital screening	Yes	Yes
Tetamman	Rest Assured	Follow-up and isolation	Yes ^1^	No
Tawakkalna	We Trust	COVID-19 health status, access public places, and electronic permits for movement, gathering, and work	Yes	No
Tabaud	Social Distancing	COVID-19 contact notification	No ^2^	No

^1^ During the time the study was conducted. ^2^ This app is usually linked and integrated with “Tawakkalna”.

**Table 2:** Tweet counts from network data after manual sentiment annotation.

mHealth App	Tweet Sentiment					Total (%)
	Positive (%)	Neutral (%)	Negative (%)	Indeterminate (%)	Sarcasm (%)	
Sehha	7 (16.7%)	28 (66.7%)	3 (7.1%)	1 (2.4%)	3 (7.1%)	42 (0.8%)
Mawid	8 (6.3%)	89 (69.5%)	22 (17.2%)	5 (3.9%)	4 (3.1%)	128 (2.5%)
Sehhaty	8 (6.3%)	98 (77.2%)	13 (10.2%)	5 (3.9%)	3 (2.4%)	127 (2.5%)
Tetamman	164 (35.9%)	257 (56.2%)	23 (5.0%)	3 (0.7%)	10 (2.2%)	457 (9.1%)
Tawakkalna	49 (1.4%)	1292 (37.7%)	143 (4.2%)	1920 (56.0%)	22 (0.6%)	3426 (67.9%)
Tabaud	9 (1.0%)	657 (75.7%)	2 (0.2%)	189 (21.8%)	11 (1.3%)	868 (17.2%)
Total	245 (4.9%)	2421 (48.0%)	206 (4.1%)	2123 (42.1%)	53 (1.0%)	5048 (100%)

**Table 3:** Tweet counts after applying data augmentation techniques.

mHealth App	Tweet Sentiment			Total (%)
	Positive (%)	Neutral (%)	Negative (%)	
Sehha	56 (56.6%)	27 (27.3%)	16 (16.2%)	99 (2.1%)
Mawid	66 (31.0%)	87 (40.8%)	60 (28.2%)	213 (4.5%)
Sehhaty	154 (31.8%)	192 (39.7%)	138 (28.5%)	484 (10.3%)
Tetamman	69 (23.5%)	141 (48.0%)	84 (28.6%)	294 (6.2%)
Tawakkalna	523 (21.7%)	1314 (54.6%)	571 (23.7%)	2408 (51.0%)
Tabaud	385 (31.5%)	704 (57.7%)	132 (10.8%)	1221 (25.9%)
Total	1253 (26.6%)	2465 (52.2%)	1001 (21.2%)	4719 (100.0%)

**Table 4:** Comparing user relationships across mHealth networks.

Network Measures	Sehha	Mawid	Sehhaty	Tetamman	Tawakkalna	Tabaud
Nodes, *n*	76	201	464	444	3076	734
Isolates, *n* (%)	9 (11.84)	29 (14.43)	9 (1.94)	40 (9.01)	65 (2.11)	14 (1.91)
Total edges, *n*	61	206	620	504	5755	1520
Unique edges, *n* (%)	55 (90.16)	175 (84.95)	320 (51.61)	391 (77.58)	2047 (35.57)	536 (35.26)
Edges with duplicates, *n* (%)	6 (9.84)	27 (13.11)	300 (48.39)	113 (22.42)	3708 (64.43)	984 (64.74)
Self-loops, *n* (%)	11 (18.03)	47 (22.81)	41 (6.61)	99 (19.64)	361 (6.27)	171 (11.25)

*n* represents the total number per measure.

**Table 5:** Comparing mHealth networks’ properties on Twitter.

Property	Sehha	Mawid	Sehhaty	Tetamman	Tawakkalna	Tabaud
Maximum geodesic distance (diameter)	5	7	8	5	8	5
Average geodesic distance	1.3782	2.5581	2.2929	2.0330	2.1515	2.0053
Connected components, *n*	30	69	57	92	153	41
Maximum nodes in a connected component, *n*	9	38	309	149	2642	575
Maximum edges in a connected component, *n*	8	52	488	149	5158	1355
Graph density	0.0165	0.0073	0.0038	0.0037	0.0006	0.0027
Modularity	0.7992	0.7485	0.4920	0.6861	0.3419	0.3354

*n* represents the total number per property.

**Table 6:** Examples of positive, neutral, and negative tweets associated with each mHealth app.

mHealth App	Positive	Neutral	Negative
Sehha	“Sehha app is truly great, the Dr. examined me while I was at home and gave me a prescription.”	“Try Sehha app, a physician will answer you. You can have 3 consultations per month for free.”	“I am physically very tired, and I do not know why until now I have not gone to the hospital, Allah, I thought I was braver than this. Even Sehha app isn’t working.”
	“تطبيق صحه جميل الصدق فحصني وانا بالبيت وعطاني وصفه طبيه”	“افتحي تطبيق صحة وترد عليك دكتوره او دكتور معك ٣ استشارات بالشهر ومجاني”	“ انا تعبانه جسديا وواصله لمرحله كبيره ولا ادري ليه للحين مارحت للمستشفى والله احسب نفسي اشجع من كذا حتى تطبيق صحه مايشتغل”
Mawid	“By using Mawid app, things are excellent”	“You can book an appointment at the health center through Mawid app.”	“I wanted to do a swab test, and I remember searching between Mawid, Sehhaty, and Tetamman apps, I got confused by the abundance of applications one is enough.”
	“عن طريق تطبيق موعد الأمور ممتازة “	“يمكنك حجز موعد لدى المركز الصحي عبر تطبيق موعد”	“كنت بعمل مسحه واتذكر جلست ادور بين تطبيق موعد وصحتي وتطمن لخبطونا بكثرة التطبيقات واحد يكفي”
Sehhaty	“I’m astonished by @SaudiMOH amount of effort. I booked an appointment for the Corona test from Sehhaty app. The entire trip, including the test, took only 18 min. A very great thing, thank you to the Ministry of Health. Honestly, I was not aware of the facilitation, until today”	“The Minister of Health announces it at the #HIMSS20ME conference. Sehaty app will be the unified application for all services provided by the Ministry of Health”	“I have a problem logging into Sehhaty app since a week ago. The same message appears, and the information is correct ??”
	“انا منبهر من حجم جهود @SaudiMOH حجزت موعد فحص كورونا من تطبيق صحتي المشوار كامل بما فيه الفحص استغرق ١٨ دقيقة فقط شيء عظيم جدا شكرا وزارة الصحة ما كنت مطلع على التسهيلات للأمانة لأني ما جربت حتى اليوم”	“وزير الصحة يعلنها في مؤتمر #HIMSS20ME تطبيق صحتي سيكون التطبيق الموّحد لجميع خدمات وزارة الصحة”	“عندي مشكلة في تسجيل الدخول لتطبيق صحتي لمدة اسبوع نفس الرسالة تظهر والمعلومات صحيحة؟؟ “
Tetamman	“Tetamman—is an excellent app. great service, organization and accurate appointments, loyal health practitioners. May Allah protect my country and keep it well and safe.”	“Tetamman app is intended for those who have been invited to download it via text messages or through a designated authority (infected or suspected of being infected). If you don’t have the conditions listed above, your isolation is considered optional, and you have the option to use the application services or delete it”	“I was contacted to download Tetamman app, but I previously downloaded it and deleted it, now the place of isolation has changed, and the isolation days do not appear ... and the questionnaire is blank”
	“تطمن- تطبيق ممتاز.. خدمة رائعة تنظيم ومواعيد مضبوطة.. ممارسين صحيين مخلصين. حفظك الله يابلادي ودمتِ بخير وامان”	“تطبيق تطمن مخصص لمن تم دعوتهم لتحميله عبر الرسائل النصية او عبر الجهة المختصة (المصابين أو المشتبه بإصابتهم) في حال لم تكن من ضمن الشروط الواردة أعلاه يعتبر عزلك اختياري ولك الخيار في استخدام خدمات التطبيق أو حذفه”	“تم التواصل معي وافادتي بتحميل تطبيق تطمن مع العلم بانه تم تحميله سابقا وتم حذفه والان تغير مكان العزل ولا يظهر ايام العزل ... وكذلك لاستبيان فارغ.”
Tawakkalna	“The reason for the decline of the epidemic in Medina after Allah is Tawakkalna app, which was strictly applied. It is prohibited to enter any government facility or private sector unless you have the app ... If you are infected, or exposed your entry is not allowed.”	“Exposed (orange and yellow color) are converted into healthy (green color) in Tawakkalna app by the Ministry of Health after 14 days without a confirmed COVID-19 infection.”	“A painful sight when you see an elderly man, a woman, or a child leaves the health center without treatment ... why? Not having access to the internet on their mobile or not having a mobile to access the Tawakkalna app ... they do not know that there are people who can’t afford it. For most the internet is only at home.”
	“ سبب انحسار الوباء بالمدينة بعد الله هو تطبيق توكلنا تم تطبيقه بحذافيره ممنوع دخول اي منشأه حكومية او قطاع خاص الا والتطبيق معك.. واذا كنت مصاب ممنوع دخولك او مخالط ممنوع دخولك”	“المخالط (اللون البرتقالي والاصفر) يتم تحويله الى سليم (اللون الأخضر) في توكلنا من قبل وزارة الصحة بعد مرور 14 يوم وعدم ثبوت الإصابة “	“منظر مؤلم عندما ترى رجل مسن أو امرأة او طفل يخرج من المركز الصحي دون علاج ... لماذا؟ ليس لديه نت في جواله أو ليس لديه جوال اصلا بدعوى تطبيق توكلنا ... لا يعلموا أن فيه ناس عائشة بالكفاف الأغلب النت في البيت. “
Tabaud	“Do you know why everyone is so proud of you @SDAIA_SA? Because, with your effort and the perseverance of your employees, you have limited the consequences of Corona, with the grace of Allah ... and we have become the top third country in the world to implement Exposure Notification technologies.”	“Tabaud app is to assist combating the Coronavirus COVID-19, to return to normal life as soon as possible by notifying the user if they were in contact with a person who was confirmed to have the virus during the past 14 days”	“#Tabaud_app I do not like anxiety, and I expect anxiety harms human health ... We depend on Allah and from my point of view, psychological aspects must be considered in any app, especially regarding human health. How this can be possible, and there is no accurate phone device that is capable of giving an accurate location without chances of error.”
	“تعرفون ليش الجميع يفخر بكم @SDAIA_SA لأنكم بجهدكم ومثابرتكم وبهمة شبابكم وشاباتكم من أبناء الوطن حديتوا من تداعيات كورونا بتوفيق الله.. وصرنا ثالث دولة في العالم تطبيقًا لتقنيات Exposure Notification”	“تطبيق “تباعد” هو للمساعدة على احتواء فيروس كورونا كوفيد١٩ والعودة إلى للحياة الطبيعية في أقرب وقت ممكن من خلال الإشعار بمخالطة شخص تم تأكيد إصابته بالفيروس خلال الـ ١٤ يوم الماضية”	“#تطبيق_تباعدانا ما أحب القلق واتوقع القلق يضر بصحة الانسان ... توكلنا على اللهومن وجهة نظري يجب مراعاة الجوانب النفسية في اي تطبيق خصوصا ما يتعلق بصحة الانسان كيف لا وأخطاء تحديد المواقع واردة ولا يوجد جهاز هاتف دقيق يعطي دقة في تحديد المواقع لا يمكن وجود خطأ معها”

**Table 7:** The performance of the three sentiment classifiers.

Classifier	Precision	Recall	F1-Score
* SVM-AraVec	0.85	0.85	0.85
SVM-tfidf	0.84	0.84	0.84
AraBERT	0.82	0.78	0.80

* Our selected classifier.

## Data Availability

Not applicable.

## Appendix A

**Table A1:** Collected tweets summary for network analysis.

mHealth App	Keywords	Arabic Keywords Translation	Total Collected	Total Included
Sehha	تطبيق صحة	Sehha application	775	61
Mawid	تطبيق موعد	Mawid application	22,766	206
	خدمة موعد	Mawid service		
	موعد	Mawid		
Sehhaty	صحتي	Sehhaty	11,254	620
	مركز تأكد	Takkad center		
	تطبيق صحتي	Sehhaty application		
	مراكز_تأكد	Takkad centers		
Tetamman	عيادات تطمن	Tetamman clinics	608	504
	مراكز تطمن	Tetamman centers		
	عيادة_تطمن	Tetamman clinic		
	برنامج تطمن	Tetamman program		
	تطبيق تطمن	Tetamman application		
Tawakkalna	@TawakkalnaApp	-	26,003	5755
	Tawakkalnaapp	-		
	توكلنا	Tawakkalna		
	تطبيق توكلنا	Tawakkalna application		
Tabaud	@TabaudApp	-	11,802	1520
	TabaudApp	-		
	Tabaud	-		
	تباعد	Tabaud		
	تطبيق تباعد	Tabaud application		
Total			73,208	8666

## Appendix B

**Table A2:** Top accounts per mHealth app based on two measures: “Betweenness Centrality” and “PageRank”.

mHealth App	Top Accounts (Betweenness Centrality)	Account Type	Top Accounts (PageRank)	Account Type
Sehha	@tfrabiah (11) @ask_madinah1 (9) @mygovsa (6)	Minister of Health Public profile Government (Website)	@mygovsa (2.619) @tfrabiah (1.852)	Government (Website) Minister of Health
Mawid	@saudimoh (444.5) @saudimoh937 (420.5) @saudinews50 (164) @tfrabiah (53.5) @ask_madinah1 (42)	Government (Health) Government (Health Call Center) Media Minister of Health Public profile	@saudimoh937 (6.7) @saudimoh (5.034)	Government (Health Call Center) Government (Health)
Sehhaty	@saudimoh (46452) @saudimoh937 (9329) @tfrabiah (1221)	Government (Health) Government (Health) Minister of Health	@saudimoh (118.867) @saudimoh937 (14.447) @kfshrc (5.749)	Government (Health) Government (Health Call Center) Public hospital
Tetamman	@sparegions (887) @saudimoh937 (531) @saudimoh (431) @tfrabiah (183) @saudiatv (162)	Media Government (Health Call Center) Government (Health) Minister of Health Media	@sparegions (17.479) @saudimoh937 (6.436) @saudimoh (6.250) @makkahregion (6.062) @ajelnews24 (5.817) @hfrmoh (4.901) @joufhealth (4.901)	Media Government (Health Call Center) Government (Health) Government (Media) Media Government (Health) Government (Health)
Tawakkalna	@tawakkalnaapp (3483315.84) @tfrabiah (67779.41) @sdaia_sa (55246.5) @mohu_csc (43380.18) @hajministry (32998.81) @absher (32924.79) @moe_gov_sa (26310)	Mobile app Minister of Health Government (Organization) Government (Religious—Hajj and Umrah) Government (Religious—Hajj and Umrah) Government (Online services) Government (Education)	@tawakkalnaapp (1110.747) @jazanuniversity (21.890) @moe_ual (14.540) @sdaia_sa (12.332) @hajministry (8.180) @ask_madinah1 (7.180) @tfrabiah (7.1300) @mohu_csc (6.730) @kfshrc_j (6.475) @_ksu (6.276) @ask_almadinah30 (6.209)	Mobile app Public university Government (Education) Government (Organization) Government (Ministry) Public account Minister of Health Government (Religious—Hajj and Umrah) Public hospital Public university Public account
Tabaud	@tabaudapp (164419.33) @jazanuniversity (946) @kauweb (109.5) @_ksu (66)	Mobile app Public university Public university Public university	@tabaudapp (257.666) @jazanuniversity (20.757) @_ksu (6.276)	Mobile app Public university Public university

## Appendix C

**Table A3:** Examples of tweets for positive and negative sentiment cases.

Positive	Negative
Appreciation: “Thank God Tawkkalna app has been launched. I can go to the mall.” “الحمدلله فتح تطبيق توكلنا اقدر انزل للسوق”	Identified weaknesses: “I wanted to do a swab test, and I remember searching between Mawid, Sehhaty, and Tetamman apps, I got confused by the abundance of applications one is enough.” “كنت بعمل مسحه واتذكر جلست ادور بين تطبيق موعد وصحتي وتطمن لخبطونا بكثرة التطبيقات واحد يكفي”
Positive opinion: “I’m astonished by @SaudiMOH amount of effort. I booked an appointment for the Corona test from the Sehhaty app. The entire trip, including the test, took only 18 min. A very great thing, thank you to the Ministry of Health. Honestly, I was not aware of the facilitation, until today” “ انا منبهر من حجم جهود @SaudiMOH حجزت موعد فحص كورونا من تطبيق صحتيالمشوار كامل بما فيه الفحص استغرق ١٨ دقيقة فقطشيء عظيم جدا شكرا وزارة الصحة ما كنت مطلع على التسهيلات للأمانة لأني ما جربت حتى اليوم”	Issues faced with apps: “Sehhay app is not responding, the activation code to reset the password is not working, the code is not sent” “تطبيق صحتي معلق، رمز التفعيل لإعادة تعين كلمه السر لا يعمل، الرمز لا يرسل “
Trust expressions around government and healthcare practitioners: “Tetamman—is an excellent app. Great service, organization and accurate appointments, loyal health practitioners. May Allah protect my country and keep you well and safe.” “تطمن- تطبيق ممتاز. خدمة رائعة تنظيم ومواعيد مضبوطة.. ممارسين صحيين مخلصين. حفظك الله يا بلادي ودمتِ بخير وامان”	Negative opinion: “A painful sight when you see an elderly man, a woman, or a child leaves the health center without treatment ... why? Not having access to the internet on their mobile or not having a mobile to access the Tawakkalna app ... they do not know that there are people who can’t afford it. For most the internet is only at home.” “منظر مؤلم عندما ترى رجل مسن أو امرأة او طفل يخرج من المركز الصحي دون علاج ... لماذا؟ ليس لديه نت في جواله أو ليس لديه جوال اصلا بدعوى تطبيق توكلنا ... لا يعلموا أن فيه ناس عائشة بالكفاف الأغلب النت في البيت. “
	Psychological impact: “#Tabaud_app I do not like anxiety and I expect anxiety harms human health ... We depend on Allah and from my point of view, psychological aspects must be considered in any app, especially regarding human health. How this can be possible, and there is no accurate phone device that is capable of giving an accurate location without chances of error.” “#تطبيق_تباعد انا ما أحب القلق واتوقع القلق يضر بصحة الانسان ... توكلنا على الله ومن وجهة نظري يجب مراعاة الجوانب النفسية في اي تطبيق خصوصا ما يتعلق بصحة الانسان كيف لا وأخطاء تحديد المواقع واردة ولا يوجد جهاز هاتف دقيق يعطي دقة في تحديد المواقع لا يمكن وجود خطأ معها”
